# The Institutional Library

**Published:** 1915-07-03

**Authors:** 


### THE INSTITUTIONAL LIBRARY.

### The Medical Annual and the War.

The Medical Annual: A Year-book of Treatment. 1915. Thirty-third Year. (Bristol: John Wright and Sons, Ltd. Illustrated. Price 10s. net.)

Of books which may unquestionably be included in a hospital library the Medical Annual holds a foremost place. For one thing, it is so much less bulky than bound volumes of the medical journals, and much more easily referred to. This year the Annual exhibits itself in no degree less important or less bulky, despite the difficulties inseparable from the circumstances of its production in a time of great stress. True its appearance has been retarded, but its publication is an achievement of which the Editor may be proud, and the fact that not one of the contributors has broken faith, although adequate excuses could readily have been forthcoming, is evidence that we are under a special obligation to them for their sacrifices. The value of the contents is not by any means limited to the "general practitioner," who is not by a long way so omnivorous or anxious to be thought omniscient as is sometimes supposed. The usual features of this issue will be found more than equal to the previous high standard, and the notes on legal decisions, for instance, will be found of as great interest and utility as hitherto. Apart from the record of progress in medical and surgical treatment, the Medical Annual contains a serviceable directory of institutions, an official and trade directory, and a convenient list of newly published books. No one who even casually goes through the book can fail to be struck by the many practical hints contained in the articles; simple methods and tests which might otherwise have escaped notice during the year's reading. For instance, there is described a method of treating sputum, which greatly facilitates the search for the tubercle bacillus. The sputum is treated with an equal volume of saturated solution of common salt, shaken thoroughly, and allowed to stand for six hours. The surface is then skimmed with a platinum loop, and a smear made. This process requires no centrifuge, and is said to possess an accuracy of 99 per cent.

In this issue of the Annual one turns very naturally to the section dealing with Naval and Military Surgery. Naval Surgery is represented by an able article by Deputy-Surgeon-General A. Gascoigne Wildney, R.N. He gives a most interesting general survey of the work on board a fighting ship, and incidentally describes the ingenious way in which hypodermic injections are administered during an action. The medical officers are supplied with a needle-fixed hypodermic syringe fitting into a metal scabbard, which is attached to the lapel of the officer's coat. The solutions are contained in differently shaped shallow bottles closed by double caps of rubber, through which the needle of the syringe is thrust. In this way injections are not only quickly given, but may also be made in the dark, a point of some importance. It should be added that a syringeful forms a single dose. Armed with this syringe and solutions of morphine, pituitrin, ergotine, etc., a haversack containing tourniquets and dressings, and carrying an ordinary bellows spray-producer, containing a 2-per-cent. solution of iodine in alcohol, attached to his belt, the surgeon is well equipped for rendering first aid. A pair of shears for cutting clothing is a useful addition.

The article on Military Surgery is by Colonel Louis A. La Garde, U.S. Army (Ret.), and deals largely with gunshot injuries of the head. Mr. G. Lenthal Cheatle, C.V.O., has written a very practical article on antiseptics in war. He alludes to his experiment with an alcoholic solution of perchloride of mercury, the alcohol being of 80-per-cent. strength. Pure malachite green, 0.25 per cent., is added to indicate exactly where the lotion has been applied. This colouring agent is itself an antiseptic, but is non-toxic, and does not diminish the antiseptic properties of the mixture, which is best applied by means of a spray. Mr. Cheatle strongly favours, as the result of practical work, the use of perchloride of mercury. The formation of a coagulum does not, as was formerly believed, render the drug impotent, but, on the contrary, the absorbed mercury in the albuminate is a valuable antiseptic. The spray is more convenient and less wasteful than a lotion, and renders possible the use of a medium that rapidly dries.

### Lay Workers and Infant Mortality.

Infant Mortality. By Hugh T. Ashby, B.A., M.D. (Cambridge: At the University Press. 1915. Pp. 229+viii. Price not stated.)

This is one of a series of volumes dealing with the various subjects connected with public health and issued from the Cambridge University Press under the joint editorship of Dr. G. S. Graham-Smith and Mr. J. E. Purvis. The books are not intended solely or even mainly for doctors, but are directed rather towards others—professional men and laymen, who in a technical or legislative or administrative capacity are associated with the medical profession in the organisation and conduct of an efficient sanitary service. Judged from this standpoint, Dr. Ashby's book may be pronounced to be well fitted for its purpose. It is beyond question that the promptings of professional advice will not have much effect unless they succeed in educating public opinion, and on the whole this is willing to be educated. Perhaps it will never come fully up to the level favoured by the experts, and we are not quite sure that such an attainment is wholly desirable. Dr. Ashby keeps to the orthodox lines and supplies a great amount of useful information in a fashion which can be readily appreciated by his readers. Though hardly new to a medical audience his chapters are well calculated to impress others who are engaged in public health responsibilities, and this is likely, we think, to be his main success. His book is very well arranged, and is written in an easy, lucid style. Platitudes in the circumstances are perhaps inevitable, and Dr. Ashby does not escape them Neither does he always succeed in saying exactly what he intends, as, for example, when he writes, "A good, contented baby is a boon in a small house," which implies either that such an arrival is not a boon in a large establishment, or that in these the noisy variety is the more welcome. Personally, we do not think he states very successfully the professional objection to the Notification of Births Act. This is not that the doctor should be made responsible for the father's neglect to send the notification, but that he should be made responsible at all. He attends the patient as a confidential adviser, and to compel him when acting in this capacity to give information regarding his patient to outsiders is to invade the sanctity of professional obligation, and is therefore inimical to the general interest. All these, however, are small points for criticism. The book is a good one and will, without doubt, reach its goal.

### "Kill that Fly."

The Minor Horrors of War. By A. E. Shipley, Sc.D., Hon. Sc.D. Princeton, F.R.S., Master of Christ's College, Cambridge, and Reader in Zoology in the University. (Smith, Elder and Co. 1s. 6d. net.)

In war-time the prevention of disease and precautions against it become of redoubled importance, and thus Dr. Shipley, as he tells us in his Preface, has passed over many other possible minor horrors of war and chosen to confine his attention to "certain little invertebrata," such as *Anoplura*, *Cimex*, etc., whose existence nowadays compels especial attention from the danger they cause to armies and to civil life in the spreading of epidemics. Of the *Anoplura* only two, *Phthirius* and *Pediculus*, attack the human body. The author discusses the latter in its two species, *Capitis* and *Vestimenti*, and draws attention to the interesting researches made by Mr. C. Warburton at Cambridge as to the habits, breeding, and means of prevention of these pests. The importance of such work is obvious, since we know that lice are the constant accompaniment of all armies; and that *Pediculus vestimenti* is a certain carrier of typhus. The *Cimex*, although peculiarly abhorrent, is perhaps not so important, as there is no evidence to show that it is a transmitter of disease. It is, however, from its disgusting nature and its extreme tenacity of life, necessary to deal with it drastically. Bugs have been kept in captivity for more than a year without a meal. The wooden huts of some of our camps would, no doubt, afford inviting shelter for these creatures. Fumigation by hydrocyanic acid, sulphur, or benzine are methods of extermination.

The flea, much more dangerous as a proved carrier of bubonic plague in its species *Xenopsylla cheopsis*, differentiated by the Hon. Charles Rothschild at Tring, is so common that the author complains that it is not treated seriously. What is wanted is, however, an effectual remedy, for no one seems able to suggest one with the certainty of the writer of the old receipt, given in the book, who ends his quaint superstition with the words, "I know it hath proved true."

Its danger as a disease-carrier and its appalling fecundity make the fly an enemy which must and can be conquered. There is no doubt that great efforts are being made in this direction, and it is interesting to note incidentally the encouraging action of health authorities, such as Dr. Mathieson, at South Shields, and Dr. Greenhalgh, at Accrington, in issuing house-to-house warnings on the danger and destruction of flies. The Panama has controlled its mosquitoes, and we could most certainly control our flies. The researches of Dr. Monckton Copeman and Mr. E. E. Austin point to the unsolved problem as to the manner in which the interval between one fly season and the next is bridged over, and suggest this interval as the time of successful future attack. A recent letter from the trenches was shown to us the other day, in which the writer gave a vivid description of his labours, which, it seemed, had been devoted to the destruction of flies. These kept him as busy as the Germans, and their losses, he added, were equally heavy. The progress of sanitary science and camp hygiene is well illustrated by this employment of troops in the destruction of flies.

Dr. Shipley ends his book with two chapters on leeches, and writes, with doubtful advantage, "in a certain spirit of gaiety," but we could wish that several of the rather cheap witticisms with which he seeks to enliven his pages had been omitted. We hope, however, that this book, so instructive at present, will be widely read.

### Water Analysis and Purification.

A Simple Method of Water Analysis. By John C. Thresh, M.D. (Vic.), D.Sc. (Lond.), D.P.H. (Camb.). (London: J. and A. Churchill. 1915. Pp. 69. 2s. 6d. net.)

As Dr. Thresh's book has reached an eighth edition it may be considered as beyond both the praise and the blame of the reviewer. It considers particularly the position of medical officers of health for rural districts who are called upon to report on water supplies to their Local Authorities. Perhaps its most striking features are its strictly practical character and the lucidity of its explanations. In the present issue a new chapter is added on the chlorine method of water purification.

### The Dispenser's Companion.

Squire's Pocket Companion to the British Pharmacopœia. By Peter W. Squire. Second Edition. (London: J. and A. Churchill. 1915. Pp. 1,040. Price 10s. 6d.)

A book so well known to hospital pharmacists as "Squire's Companion" to the British Pharmacopœia needs no introduction, but its new form demands notice, for this handsome leather-bound volume is the result of the demand for a less bulky book than the original "Companion," which had continued to grow in size owing to the large amount of experimental work therein described. It was therefore decided in 1904 to issue a pocket edition of the work which should contain only matter of special interest to the practitioner and dispenser. By the use of thin paper the thousand odd pages occupy a space which may, with justification, be considered well within the dimensions of a pocket. It is, we are sure, no exaggeration to describe this book as being more widely disseminated among hospital dispensaries and doctors' surgeries than the British Pharmacopœia itself. The now almost extinct dispenser-house-surgeon would scarcely dare to be without it, and of its practical value as a reference book to the general practitioner there is surely no need to speak. This pocket edition omits nothing which is pertinent to such matters as commonly arise in the ordinary course of prescribing and dispensing. It is a systematic and exhaustive review of the alterations and additions found in the British Pharmacopœia of 1914. The popularity of this "Companion" has always been largely due to the concise information given about non-official drugs and preparations. In this edition the non-official matter is exceedingly well represented, and there can be no new drugs of any importance about which this volume is silent. To medical men the extracts from the literature bearing on the therapeutic value or otherwise of individual preparations will be of value, though in many cases it must be borne in mind that results reported in medical journals are not always implicitly to be relied upon. Among the more important monographs must be noted the full description of the use of salvarsan and the methods of its administration. The special chapter on therapeutic agents of microbial origin has been revised and rewritten to a large extent by Professor Tanner Hewlett; it contains some useful information on serums, vaccines, tuberculins, and the like. The section giving a classification of remedies on a therapeutic basis is likely to be often used, but the following section, which gives lists of remedies employed in special ailments, is one the utility of which is not so obvious. In a work of this description the fulness of the index is of paramount importance, and it is pleasing to record that no falling off has been detected in this respect in the pocket edition.

